# A rare case of colorectal cancer metastasis to the pancreas: a case report

**DOI:** 10.1093/jscr/rjae173

**Published:** 2024-03-26

**Authors:** Hesameddin Eghlimi, Moein Mirzadeh, Hamidreza Movahedi, Tala Tabrizi

**Affiliations:** Department of General Surgery, School of Medicine, Ayatollah Taleghani Hospital, Shahid Beheshti University of Medical Sciences, Tehran 19857-17443, Iran; Department of General Surgery, School of Medicine, Ayatollah Taleghani Hospital, Shahid Beheshti University of Medical Sciences, Tehran 19857-17443, Iran; Department of General Surgery, School of Medicine, Ayatollah Taleghani Hospital, Shahid Beheshti University of Medical Sciences, Tehran 19857-17443, Iran; Department of General Surgery, School of Medicine, Ayatollah Taleghani Hospital, Shahid Beheshti University of Medical Sciences, Tehran 19857-17443, Iran

**Keywords:** colon cancer, metastasis, pancreas

## Abstract

Colon cancer is the third leading cause of cancer worldwide. On presentation, 20% of patients will have metastatic disease, and the most common sites for metastatic colon cancer are liver, lung, and peritoneum. Our patient was a 55-year-old man with a history of rectal adenocarcinoma cancer and colectomy surgery for the past 2 years, who had presented changes in bowel movements, rectal bleeding and thin. In the new visit and positron emission tomography-computed tomography scan, the results showed that there are two ill-defined foci of increased FDG (F-fluorodeoxyglucose) uptake in the body (metabolic diameter = 26 mm, SUVmax = 7.8) and tail (metabolic diameter = 13 mm, SUVmax = 7.4) of the pancreas gland, persisting on delayed images. Colon cancer metastasis to the pancreas is rare. The presence of masses in both the colon and pancreas could be a result of metastasis from the pancreas to the colon, metastasis from the colon to the pancreas, or synchronous primary cancers.

## Introduction

Colorectal cancer (CRC) is the third most common cancer in the world. Approximately 56% of patients with CRC die of their cancer. The occurrence of metastasis is a major concern for patients and doctors because metastasis may be fatal and cause a mass effect and interfere with the patient’s homeostasis [1]. Approximately 20% of patients with CRC already have metastases at diagnosis. With continued advances in CRC treatment, survival rates have improved. Under appropriate conditions, the median survival in patients with solitary lung or liver metastases may exceed 5 years [2]. Early diagnosis of CRC is important and necessary due to the negative impact of metastatic cancer on survival. Although current evidence has shown a reduction in cancer-specific mortality from CRC in screening cohorts, all-cause mortality may not improve with screening.

The development of studies in metastasis research has significantly expanded the knowledge of metastasis at the cellular and molecular level [3]. Unfortunately, the level of epidemiological knowledge in this field is low. Efforts to investigate metastases are hampered by the fact that cancer registries rarely include information on metastases separately from the stage of diagnosis. In this situation, it is impossible to assess metastatic spread to specific sites. An overview of metastatic patterns in various cancers is limited to autopsy-based studies that rely on about a thousand deaths from metastatic cancer [4].

## Case presentation

Metastatic carcinoma of the pancreas from another primary site is uncommon and it accounts for 2%–5% of all pancreatic cancer cases [5]. Investigating the epidemiology of metastatic CRC is challenging because cancer registries rarely record metastatic sites. It has been reported that 5%–10% of all pancreatic cancers are estimated to be attributable to inherited risk factors and some patients who had no family history of this cancer harbor at least one known.

There are a variety of cancer types which have been shown to metastasize into the pancreas as mass lesions, such as renal cell carcinoma (RCC), lung cancer, colon rectal cancer, breast cancer, liver, ovary, urinary bladder, prostate, uterus, Merkel cell carcinoma, lymphoma, and melanoma [7].

**Figure 1:**
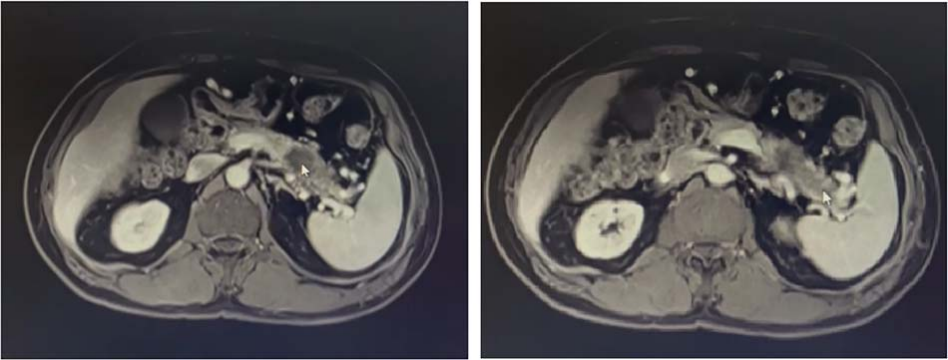
Metastatic masses can be seen in the pancreatic tissue on the CT-Scan (transverse/axial view)

**Figure 2:**
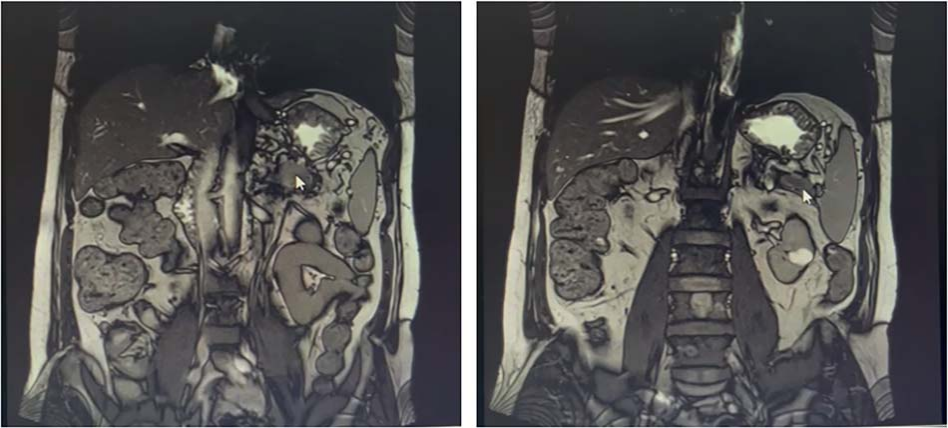
Metastatic masses can be seen in the pancreatic tissue on the CT-Scan (coronal view)

Our patient is a 55-year-old man with a history of rectal adenocarcinoma cancer in the last 2 years. At that time, he presented with flank pain and kidney stones. After examination and diagnostic tests, he was diagnosed with colon cancer that a 12 cm mass was reported and then colectomy surgery was performed. Then six sessions of radiotherapy were considered for him. In the new regression, 2 years after the colectomy surgery, during the examination phase, due to the exacerbation of pain, a computed tomogaphy (CT) scan (Figs 1 and 2*)* and endoscopy were requested for the patient, in which pancreatic cancer was observed. After that, the patient was placed under endoscopic ultrasound (EUS), which is reported. There was one 35 × 29 mm hypoechoic and round lesion with ill-defined border in pancreatic tail with no involvement of pancreatic duct or splenic vessels in favor of metastasis. EUS fine-needle biopsy with needle 22G was performed. Then the patient underwent a biopsy and the final confirmation was made.

### Investigations

The result of the patient’s pathology report in his first visit was as follows: on opening the lumens shows an ulcerated creamy-gray mass measuring:

(1) 1.5 × 1.5 × 0.7 cm with 4 cm distance from distal margin. Two lymph nodes were identified. SOS: M/4 & Embedded: 1%.

(2) Consists of one fragment of creamy-gray irregular tissue measuring:2 × 1.5 × 1.5 cm. Labeled as proximal margin of colon. SOS: M/2 & Embedded Totally.

(3) Consists of one fragment of creamy-gray irregular tissue measuring: 1.5 × 1 cm. Labeled as distal margin of colon. SOS: M/1 & Embedded Totally.

The final diagnosis presented in the pathology report showed that colon, rectosigmoid segment had well-differentiated adenocarcinoma.

In the spiral CT scan of the lung, abdomen, and pelvis with and without injection for the patient, the results showed that the image of a nodule with a spicule border with a diameter of 10 mm was evident in the right area along with subsegmental atelectasis, and the evidence of two faint opacities in left lower lobe and right lower lobe. The right area was in the place of turbidity.

FDG-PET/CT Scan (videos 1 and 2) on abdominal and pelvis reported that calcified granuloma without FDG uptake was seen in the liver segment II. There were two ill-defined foci of increased FDG uptake in the body (metabolic diameter = 26 mm, SUVmax = 7.8) and tail (metabolic diameter = 13 mm, SUVmax = 7.4) of the pancreas gland, persisting on delayed images. Hypermetabolic tumoral lesion in the body and tail of the pancreas are suggestive of metastasis.

## Discussion

Tumors (also called neoplasms) are masses of cells. They can be benign (not cancer) or malignant (cancer). Metastasis of pancreatic cancer to the colon is extremely rare, with <10 cases reported in the literature. In this article, we reported the case report of a 55-year-old man who had metastasized to the pancreas 2 years after being diagnosed with CRC.

The diagnosis of cancer metastasis to the pancreas should be suspected when the patient has a history of malignancy, especially one of kidney, skin, lung, colon, or breast cancer [8]. Besides imaging study, such as CT, bone, and PET/CT scan, EUS-guided biopsy has a most important value for ruling out a second primary pancreatic cancer. The prognosis of pancreatic metastases is essentially determined by the underlying primary cancer and the potential treatment options [8]. Compared with pancreatic metastases from RCC, metastases from melanoma and lung cancer were associated with worse survival [9]. Patients with metastatic colon cancer have a relatively higher survival rate because of the availability of multiple chemotherapy agents. If they are unable to undergo chemotherapy, their co-morbidities might contribute to early death.

It has been reported that 5%–10% of all pancreatic cancers are estimated to be attributable to inherited risk factors and some patients who had no family history of this cancer harbor at least one known inherited pancreatic cancer-predisposing genetic alteration [6].

Most of the malignant lesions of the pancreas are primary tumors of the pancreas and show only a small percentage of metastasis. When pancreatic malignancies become symptomatic, they often present at an advanced stage with obstruction of the pancreatic ducts and/or bile ducts [10]. The size, location, and spread of the lesion have a significant impact on available treatments and prognosis. New-onset diabetes mellitus is associated with pancreatic adenocarcinoma, although the exact mechanism of this relationship is unknown. Proposed mechanisms include a paraneoplastic effect of pancreatic adenocarcinoma, islet function modulating inflammation, and a lack of pancreatic polypeptide response [11].

Although pancreatic metastases are rare, the most common tumor that metastasizes to the pancreas is RCC [12]. Although the patient under study and under our treatment had kidney stones for several years, this condition can be related to the problems in the kidney cells. Management of pancreatic malignancies due to metastatic disease depends on the source of the primary disease and the extent of other metastatic disease. RCC is more likely to metastasize the pancreas, so surgery may be an option. As we saw in our patient, reports of other primary malignancies that spread to the pancreas (colon) often have metastatic disease requiring systemic therapy. Prognosis in patients with pancreatic metastasis is directly related to the type of primary malignancy. Metastatic RCC, breast cancer, and colon cancer have higher survival rates than metastatic sarcoma and lung cancer.

## Supplementary Material

video_1_rjae173 video 1: Slices or cuts of the scan are shown in a video confirming metastatic masses which are seen in the pancreatic tissue on the CT-Scan (transverse/axial view)

video_2_rjae173 video 2: Slices or cuts of the scan are shown in a video confirming metastatic masses which are seen in the pancreatic tissue on the CT-Scan (coronal view)
